# 4308 DEEP-PRIMED IL-15 SUPERAGONIST IMPROVES ANTIVIRAL EFFICACY OF HIV-SPECIFIC CD8^+^ T-CELLS IN HUMANIZED MOUSE MODEL

**DOI:** 10.1017/cts.2020.59

**Published:** 2020-07-29

**Authors:** Chase Daniel McCann, Elizabeth Zale, Adam Ward, Thomas Dilling, Ali Danesh, Eva Stevenson, Talia Mota, Austin Boesch, Thomas Andresen, Darrell Irvine, R. Brad Jones

**Affiliations:** 1 Clinical and Translational Science Center, Weill Cornell

## Abstract

OBJECTIVES/GOALS: HIV-specific CD8^+^ T-cells play a critical role in partially controlling viral replication in infected-individuals, but ultimately fail to eliminate infection. Enhancing these T-cell responses through lymphocyte engineering approaches has the potential as a novel therapy capable of achieving durable control or eradication of infection. METHODS/STUDY POPULATION: IL-15 Superagonist (IL-15SA) potently supports the *in vivo* persistence and antiviral activity of adoptively transferred CD8^+^ T-cells. The Deep-Priming^TM^ technology platform, developed by Torque, allows for loading of immunomodulators onto the surface of T-cells via electrostatic ‘nanogels’, which slowly release to deliver sustained autocrine immune stimulation without the harmful effects of systemic exposure. Here, we investigate the impact of IL-15SA Deep-Priming on HIV-specific CD8^+^ T-cells in a humanized mouse model of HIV infection. Humanized mice were generated by engrafting NOD-*scid*-IL2Rg^null^ mice with memory CD4^+^ T-cells isolated from an ARV-suppressed HIV+ donor. An autologous HIV-specific Cytotoxic T-Lymphocyte (CTL) clone was isolated, and killing potential confirmed. Four weeks post humanization, mice were infected with HIV and received an infusion of unmodified HIV-Specific CTLs, or IL-15SA Deep-Primed HIV-specific CTLs (CTL-DP). T-cell numbers and plasma viral loads were quantified weekly by flow cytometry and qRT-PCR. RESULTS/ANTICIPATED RESULTS: Mice receiving unmodified CTLs trended toward reduced viral loads compared to the No Treatment condition, while mice receiving CTL-DP saw significant, 2-Log_10_ reductions in VL (p < 0.01). At 41 days post-infection 100% (5/5) of the No Treatment, 66.7% (4/6) of the CTL treatment, and 16.7% (1/6) of CTL-DP treatment mice had detectable viremia. IL-15SA Deep-Priming increased CTL expansion and persistence in peripheral blood which correlated with improved CD4^+^T-cell preservation. DISCUSSION/SIGNIFICANCE OF IMPACT: Here we demonstrate the first *in vivo* analysis of IL-15SA Deep-Priming of HIV-Specific CTLs. These data suggest that Deep-Priming of patient T-cells can enhance *in vivo* function and persistence, leading to improved viral suppression; a significant advancement in the field of HIV cure research. CONFLICT OF INTEREST DESCRIPTION: Austin Boesch, Thomas Andresen, and Douglas Jones are employees of Torque. Darrell Irvine is a co-founder of Torque and Chairman of Torque’s Scientific Advisory Board.

